# Impact of udder infections on biochemical composition of milk in context of pesticides exposure

**DOI:** 10.14202/vetworld.2022.797-808

**Published:** 2022-03-31

**Authors:** Hala R. Ali, Samah F. Ali, Rania H. Abd-Algawad, Fayza A. Sdeek, Mahmoud Arafa, Essam Kamel, Momtaz A. Shahein

**Affiliations:** 1 Bacteriology Department, Animal Health Research Institute, Agriculture Research Center, Giza, Egypt; 2 Mycoplasma Department, Animal Health Research Institute Agriculture Research Center, Giza, Egypt; 3 Pesticide Residue Department, CAPL, Agriculture Research Center, Giza, Egypt; 4 Biochemistry and Toxicology Department, Animal Health Research Institute, Agriculture Research Center, Giza, Egypt; 5 Department of Virology Research, Animal Health Research Institute, Agriculture Research Center, Giza, Egypt

**Keywords:** antioxidant bioactivities, mastitis, *Mycoplasma* mastitis, organochlorine pesticide

## Abstract

**Background and Aim:**

Environmental contaminants such as pesticides have shown immunomodulatory effects that can make animals highly susceptible to pathogenic invasion. The current work aims to study the incidence of udder infections in a single dairy herd of 160 cows in Qalyoubia Governorate, in relation to the potential intoxication of dairy cattle with organochlorine (OCs) pesticides. The study also aims to investigate the impact of udder infections on milk composition.

**Materials and Methods:**

The dairy herd was screened for udder infections using the California mastitis test and measurement of somatic cell count (SCC), followed by bacteriological and molecular analysis. In parallel, the milk samples were also tested for residues of 15 OCs compounds using gas chromatographic analysis.

**Results:**

The examined herd showed a high prevalence of mastitis (37.5%) and *Mycoplasma* was identified as the main bacterial pathogen. OCs residues were detected in milk of 45 cows out of 160 with a higher incidence in mastitic (43.3%) than in healthy cows (19%). Further, the biochemical analysis of milk showed a significant drop in major electrolytes combined with a significant rise in blood-borne electrolytes (Na and Cl) and total protein. This was more extreme in the case of *Mycoplasam* mastitis compared to non-*Mycoplasma* mastitis. In addition, *Mycoplasma* mastitic milk revealed a high level of malondialdehyde associated with reduced antioxidant enzymes (glutathione peroxidase, superoxide dismutase and catalase), compared to non-*Mycoplasma* mastitis.

**Conclusion:**

*Mycoplasma* mastitis was shown to be associated with increased SCC and, in turn, appeared significantly correlated with increased biochemical changes in milk, indicating the serious impact of *Mycoplasma* mastitis on the dairy industry. Our data also show a strong correlation between increased SCC and biochemical changes in milk, suggesting that tested biochemical parameters might serve as potential biomarkers for the early detection of mastitis. The study also suggested a potential relationship between poisoning with OCs and susceptibility to bacterial udder infections. However, further studies are required to examine the immune status of a dairy herd in relation to the level of OCs in cow’s blood, as well as the water sources used, grass forage and soil.

## Introduction

Bovine mastitis or udder inflammation is a serious problem in dairies worldwide. It is associated with severe economic losses due to reduced milk production and quality, increased culling rates, and high treatments or preventive costs [1-3]. Various bacterial pathogens such as *Staphylococcus aureus*, *Streptococcus agalactiae*, and *Escherichia coli* caused bovine mastitis. In addition, *Mycoplasma* mastitis has increased in many countries [4,5], and represents a growing concern in many dairies in Egypt. The ability of this pathogen to survive and persists in dairy farms under humid environmental conditions contributes to the dynamic spread of infection [6]. Many peer-reviewed articles have already discussed the risk factors associated with udder inflammation caused by *Mycoplasma* or other pathogens, such as introducing new un-tested animals to poor herd ventilation or unhygienic milking practices [7]. However, predisposing factors that may make the herd more vulnerable to develop udder infections have not been sufficiently investigated. Pesticide exposure is one of the significant predisposing factors for pathogen invasion and susceptibility to infectious diseases [8]. Pesticides are synthetic organic compounds that are widely used in agriculture to kill unwanted weeds and control pests [9]. Organic pesticides are used extensively as insecticides in livestock and crop protections and include organochlorine (OCs), the most hazardous substances released in the ecosystems. The high lipophilic and poor degradable nature of OCs, allow them to bioaccumulate in living organisms. The extensive use of compounds prone to bioaccumulation facilitates its dissemination in the environment and increases the risk of livestock exposure by ingestion or through the skin. The potential harm posed to animal health, welfare, and productivity is augmented by the risk of pesticides transfer to animal products (e.g., milk and meat).

Contaminated surface and groundwater serve as a major way for pesticides intoxication in cattle [10]. In 2005, the Sacco River in Rome was contaminated with OCs pesticides, putting many nearby dairy farms at risk of OCs exposure. The authors in a case report uncovered contamination of farm soil associated with a high level of OCs in cattle blood and milk, which poses a serious health hazard to humans and animals [11]. A recent study in Egypt reported residues for seven OCs pesticide compounds in irrigation water and various water sources. However, the use of these compounds was prohibited a long time ago [12]. The existence of OCs in water sources could be attributed to their persistent nature, increasing the danger of Egyptian livestock being exposed. Dairy cattle are already at high risk of stress due to labor and lactation; hence, their exposure to pesticides may make them more vulnerable to pathogenic infection and diseases, especially mastitis. Pesticides can target innate and adaptive components of the immune system, leaving the affected organism unprotected against pathogens invasion [13]. The outcome of udder invasion depends on the pathogenicity of the involved bacterial species and tolerance of the cow’s immune system. Compromising the immune competence of lactating cows leads to an increased risk of udder invasion by pathogenic organisms. Lactating dairy cows are generally affected by various factors that are related to host or pathogens, including genetic, physiological, and environmental factors, reducing the host’s immunity and increasing their susceptibility to udder infections [14].

Inflammatory udder diseases are associated with modification of milk composition [15]; this is mediated by the severity of infection, virulence of the causative agents and extent of secondary physiological disturbance, which impair the normal function of udder [16]. Studying the biochemical components in milk, in relation to the pathogens that provoke mastitis, may further our understanding of the pathogens involved and ultimately help identify their biomarkers. Kayano *et al*. demonstrated that changes in milk composition were varied based on the causative agent, showing that *Streptococcus* spp. and coliforms appeared to be associated with long-term fat, protein, and milk urea changes, while decreased lactose was found associated with *S. aureus* mastitis [17]. The impact of conventional mastitis pathogens, including *S. aureus*, on milk composition has been widely investigated [18]; however, the effect of *Mycoplasma* mastitis on milk composition is not sufficiently clear.

The present work studied the impact of udder infections on the biochemical composition of milk with reference to the incidence of pesticides residues in a single herd of 160 cows in Egypt. We argue that pesticides exposure might be a major predisposing factor in the growing incidence of udder infections in the examined dairy herd. Within the diagnosed herd mastitis was characterized by a high somatic cell count (SCC) that appeared to be associated with positivity to *Mycoplasma* and in turn, consistently correlated with increased biochemical changes in milk, compared to non-*Mycoplasma* mastitis. This suggests the negative effect of *Mycoplasma* mastitis on the dairy industry.

## Materials and Methods

### Ethical approval

Ethical approval was not required as the milk samples were collected during daily routine inspection of milking.

### Study period and location

The study was conducted during January 2019 in Qalyoubia Governorate, Egypt.

### Preparation of standard

Stock solution preparation, 100 mg/mL reference standard solutions of examined pesticides (Augsburg, Germany) were prepared in 100 mL hexane (Scharlau, Barcelona, Spain) in a volumetric flask. Stock solutions were kept in a refrigerator at 4°C. Preparation of 10 mg/mL individual standards for each pesticide was achieved by diluting 5.0 mL of stock solution in 50 μL hexane. The intermediate solution was maintained in the refrigerator at 4°C. Calibration standards of OCs mixture were also achieved by dissolving appropriate amounts of intermediate solutions in hexane and matrix extract.

### Sample collection

Milk samples were drawn under the aseptic conditions from healthy dairy cows and cows suffering from mastitis in Qalyoubia Governorate immediately before routine daily milking. The udder of each cow was washed with a diluted solution of povidone-iodine (El-Nile Co., Egypt) in warm water, dried with paper towels, and teats were swabbed with 70% ethyl alcohol and allowed to air dry for 1 min. Milk samples (150 mL) were drawn aseptically from each quarter directly into labeled with Whirl-pak sampling bags (28 g, Merck Darmstadt, Germany); these were placed in iced insulated boxes. Samples were examined for *Mycoplasma* by the traditional golden isolation method and polymerase chain reaction (PCR) test [19].

For chemical and pesticide analysis, samples were packaged in glass tubes (Merck). All samples were maintained at −10°C until analyzed.

### California mastitis test (CMT)

The test conducted as described by Kandiwa *et al*. [20]. Results were interpreted referring to the change in color and grade of gel formation as negative, trace, 1+, 2+, and 3+ as described by Kibebew *et al*. [21].

### Isolation and biochemical characterization

Milk samples were inoculated on Pleuro Pneumonia Like organisms (PPLO) broth, the medium was then plated on PPLO agar medium (HiMedia, India) [22], incubated at 37°C for 3-7 days with 24-48 h observation interval for the characteristic *Mycoplasma* “fried egg” colonies.

Biochemical identification of the isolates was performed according to Hazelton *et al*. [23]. Digitonin sensitivity, glucose fermentation, arginine deamination, and film and spot formation tests were applied as mentioned by Citti *et al*. [24].

### PCR

DNA extraction used a Thermo genomic DNA extraction kit, Cat. No. k0721, (Thermo Fisher Scientific, Vilnius, Lithuania), was used as described by the manufacturer. PCR reactions were performed in a Gradient Thermal cycler 1000S (Bio-Rad, USA). The reaction mixture (total volume of 50 μL) was 25 μL Dream Green PCR Mix (Dream Taq Green PCR Master Mix (2X) Thermo Scientific), 5 μL target DNA, 2 μL of each primer (Table-1) [25,26], (containing 10 pmole/μL) and the mixture was completed by RNAse DNAse free sterile distilled water to 50 μL. PCR amplification of 16S-23SrRNA gene and 16S rRNA gene carried used the thermal profile described previously by Matucci *et al*. [25] and Peng *et al*. [26], respectively. Further, the presence of amplified PCR products was confirmed using a 1.5% agarose gel, followed by ultraviolet visualization after ethidium bromide staining (Thermo Fisher Scientific) [25,26].

**Table-1:** Primers sequences used for molecular identification of *Mycoplasma* spp. and *Mycoplasma bovis*.

Mycoplasma spp.	Target gene	Primer sequence (5′→3′)	Amplicon Size (bp)	Reference
*Mycoplasma* spp.	*16S-23SrRNA*	GGT GAA TAC GTT CTC GGG TCT TGT ACA CAC	600-1000	[25]
		CTT TTC ACC TTT CCC TCA CGG TAC		
*Mycoplasma bovis*	*16SrRNA*	CCT TTT AGA TTG GGA TAG CGG ATG	360	[26]
		CCG TCA AGG TAG CAT CAT TTC CTA T		

### Biochemical milk studies

The concentration of sodium (Na), potassium (K), calcium (Ca), and magnesium (Mg) in milk samples was determined with an atomic absorption spectrophotometer (B3003, Perkin Elmer-AAS, MA, USA). Samples were prepared according to the procedures described in the technical manual of the AAS. The data obtained were expressed as mg/dL. Milk phosphorus (P) determination was performed according to the technique of Gliszczyńska-Świgło and Rybicka [27] and chloride (Cl) was performed according to Jaudzems *et al*. [28].

Analysis of malondialdehyde (MDA) was conducted using ELISA technique (ELISA kit, Cusabio-, China), glutathione peroxidase (GSH) concentration by the modified method of Tan *et al*. [29] catalase enzyme activity according to Moretti *et al*. [30] and total antioxidant according to method described by Yagi [31].

Determination of total protein in milk was performed according to the method described by Buzanovskii [32]. Superoxide dismutase (SOD) activity was measured following the method proposed by Camkurt [33].

### Pesticide residual analysis

#### Extraction procedure [34]:

Milk samples were thoroughly mixed, followed by transferring 5 mL of homogenate in 50 mL PFTE tube containing a mixture of 10 mL deionized water and 10 mL acidified acetonitrile with acetic acid (Scharlau, Barcelona, Spain). The mixture was shaken manually, then added 4.0 g of anhydrous Mg sulfate, 1.0 g of Na Cl, 1 g of Na citrate, and 0.5 g Na hydrogen citrate sesquihydrate (Merck, Darmstadt, Germany). Finally, the content was mixed by shaking for 1 min, followed by centrifugation at 1792 × g for 3 min. Subsequently, 1 mL of the supernatant clear solution was transferred into 15 mL of polyethylene tube containing 25 mg amine sorbent (Supelco, Bellefonte, USA) and 150 mg anhydrous Mg sulfate (Merck). Then the tube was shaken vigorously manually for 1 min then centrifuged (Sigma, Darmstadt, Germany) for 1 min. at 2800 ×g. Finally, 0.5 mL of supernatant was taken into a glass vial, evaporated to dryness, and re-dissolved in 0.5 mL n-hexane for gas chromatograph-microelectron capture detector (GC-μECD) analysis (Agilent Technologies, Santa Clara, CA, USA).

### GC analysis

OCs pesticides were analyzed using Agilent 7890, GC system (Agilent Technologies, Santa Clara, CA, USA), equipped with electron capture detector (GC-μECD). GC investigation was conducted using HP-5 MS capillary column of 30 m, 0.25 mm Id., and 0.25 μm film thicknesses. The temperature of oven was adjusted 80°C for 1 min, increasing to 30°C/min up to 160, then to 260°C at a rate of 3°C/min. Temperature was maintained at 260°C for 12 min. The temperature of the injector and detector were maintained at 300 and 320°C, respectively. Carrier gas (nitrogen) was used at a flow rate of 3 mL/min. Each set of samples was analyzed against solvent blank, standard mixture and procedural blank were run in sequence to check for contamination. The chromatograph peaks were then identified and quantified. Selected samples were analyzed by full scan GC–MS to confirm the GC-μECD results. An Agilent System 6890 series plus GC, equipped with an Agilent 5973 mass selective detector, an autosampler of Agilent 7683 series and a split/splitless capillary injector port, was employed. The column used was HP-5 MS (Agilent, Folsom, CA, USA) capillary column of 30 m, 0.25 mm id., 0.25 mm film thickness. Helium was the carrier gas at a flow rate of 0.5 mL/min. The injection volume was 1 mL and the inlet temperature was fixed at 225°C. The oven temperature was 60°C (2 min) followed by a gradual increase of 10°C/min until reach to 160°C and finally rise 3°C/min until reach to 260°C (20 min). The helium carrier gas flow rate was kept in a constant flow mode of 1.0 mL/min. Sample injection was carried out split less at 240°C with 1 min purge off. The GC–MS interface was 280°C.ChemStation software (https://www.agilent.com/en/product/software) was used for instrument control and data analysis.

### Method of validation

The validation technique was carried out according to the Analytical Quality Control and Method Validation Procedures for Pesticide Residues Analysis in Food and Feed, document 10684/2009 (SANCO/10684/2009). First, linearity was calculated by constructing matrix-matched calibration curves in a range of 0.1-20 μg/L for GC-μECD. Sensitivity and recovery % were determined using spiked samples with the tested pesticides at three different levels. As stated in Table-2, the average recovery % for fortified samples was calculated. Limits of detection and limits of quantification were estimated according to pesticide concentration produced a peak signal-to-noise ratio of 3/1 and 10/1, respectively.

**Table-2:** Recovery Percentage from fortified milk samples and the minimum detection limits (μg/kg) for tested pesticides.

Pesticide name	Recovery	RSD	LOD	LOQ	r^2^
HCB	87	2.1	0.62	1.43	0.991
a-HCH	101	11	0.003	0.005	0.989
c-HCH	95	9	0.002	0.003	0.994
d-HCH	96	10	0.003	0.005	0.992
Aldrin	87	10	0.002	0.004	0.993
Endrin	105	14	0.001	0.004	0.992
Dieldrin	101	12	0.001	0.005	0.991
Heptachlor	95	10	0.003	0.006	0.990
Hept. Epoxide	101	15	0.001	0.004	0.992
c-Chlordane	91	9	0.003	0.005	0.984
Endosulfan	93	13	0.001	0.006	0.991
p, p0 –DDE	99	9	0.001	0.004	0.992
p, p0 –DDD	92	12	0.002	0.005	0.992
p, p0 –DDT	96	14	0.001	0.006	0.993
Methoxychlor	92	6	0.002	0.004	0.991

LOD=Limits of detection, LOQ=Limits of quantification, RSD=Relative Standard Deviation OCP=Organochlorine pesticides, DDE=dichlorodiphenyldichloroethylene DDD=Dichlorodiphenyldichloroethane, DDT=Dichlorodiphenyltrichloroethane, HCB=Hexachlorobenzene, HCH=Hexachlorocyclohexane

### Statistical analysis

Data obtained were statistically analyzed using repeated-measures analysis of variance. The significant main effects were detected using the least significant difference for multiple comparisons. The level of significance was at p<0.005 using IBM-SPSS Version 20 [35].

## Results

### Incidence of mastitis in the dairy herd

To determine the incidence of mastitis in a herd of 160 lactating cows located in Qalyoubia Governorate, Egypt. The cows were first screened for mastitis using CMT, followed by a measurement of SCC. Fifty-two dairy cows (32.5%) were found positive for CMT with high SCC ranged from 600 K to >1500 k while 5% of examined cows identified in a subclinical phase of mastitis based on positive CMT and SCC range of 300-500 K. Overall, the incidence of clinical and subclinical mastitis in the single examined herd registered 37.5%. Cows with SCC <300 K and negative CMT were considered as healthy.

Furthermore, the milk samples from dairy cows with positive CMT and SCC over 300 K were primarily subjected to bacteriological analysis to detect the pathogens that trigger udder inflammation in the examined dairy herd. The result identified *Mycoplasma* as the main pathogen causing clinical and subclinical mastitis, where 32.5% (52) of the samples were found bacteriologically positive for *Mycoplasma*, showing the culture characteristics for *Mycoplasma* including digitonin sensitive colonies with dark centers and typical fried-egg appearance. Biochemical analysis characterized 41 isolates as *Mycoplasma bovis* with negative glucose and arginine and positive reaction for film and spot formation.

**Figure-1:**
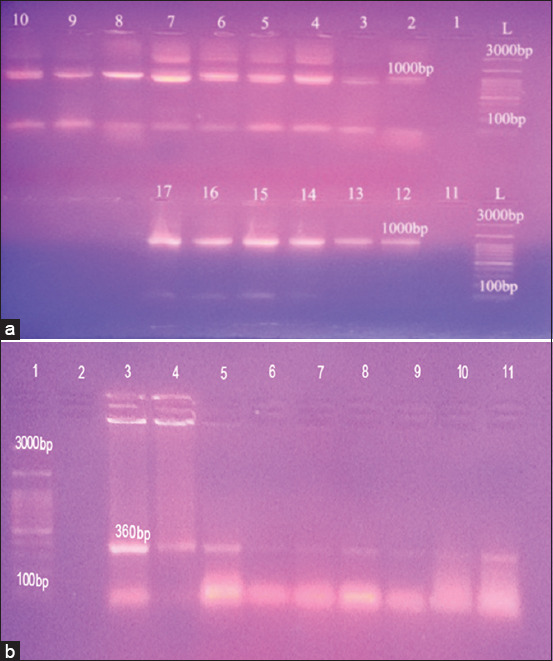
Molecular characterization of *Mycoplasma* spp. and *Mycoplasma bovis*. (a) Agarose gel electrophoretic pattern of 16S-23SrRNA gene of Mycoplasma. L (Lader): 100 bp DNA ladder, Lanes (1, 11): Control negative, Lanes (2, 12): Control positive, Lanes (3-10): Positive isolates at 1000 bp and Lanes (13-17): Positive isolates at 1000 bp. (b): Agarose gel electrophoretic pattern of 16SrRNA specific gene of *M. bovis*: Lane (1): 100 bp DNA ladder, Lane (2) Control negative, Lane (3): Control positive and Lanes (4-11): Positive isolates at 360bp.

16S-23SrRNA based PCR is furtherly applied to confirm the identity of the bacteriological positives *Mycoplasma* isolates, revealing that all the 52 *Mycoplasma* isolates showed a characteristic band at 1000 bp (Figure-1a and Table-3). Among the 52 *Mycoplasma* isolates, 41 were detected positive for 16SrRNA specific gene for *M. bovis* with a distinct band at 360 bp (Figure-1b and Table-3).

**Table-3:** Incidence of *Mycoplasma* positive and *Mycoplasma* negative mastitis in a single dairy herd linked to SCC.

SCC×10^8^	*Mycoplasma* mastitis		Non*-Mycoplasma* mastitis

	*Mycoplasma bovis*	*Mycoplasma* spp.	
<3	-	-	-
3-5	-	-	8 (5%)
6-8	5	1	-
9-15	8	4	-
>15	28	6	-
Total	52 (32.5%)		8 (5%)

SCC=Somatic cell count

As indicated in Table-3, milk with high somatic cell content >15 K was found infected with *Mycoplasma* especially, *M. bovis*, while milk samples with the lower SCC tested negative for *Mycoplasma*.

### Biochemical analysis of normal and mastitic milk in the examined dairy herd

We investigated the impact of *Mycoplasm*a mastitis compared to non*-Mycoplasm*a mastitis on the biochemical composition of milk. Milk samples from the examined dairy herd were divided into five groups according to the SCC and *Mycoplasma* positivity as follow: Group A: Negative mastitis (SCC<300 K), Group B: Non-*Mycoplasma* mastitis (SCC: 300 K-600 K), Groups C, D, and E: *Mycoplasma* mastitis (SCC: 800 K up to >1500 K).

**Figure-2:**
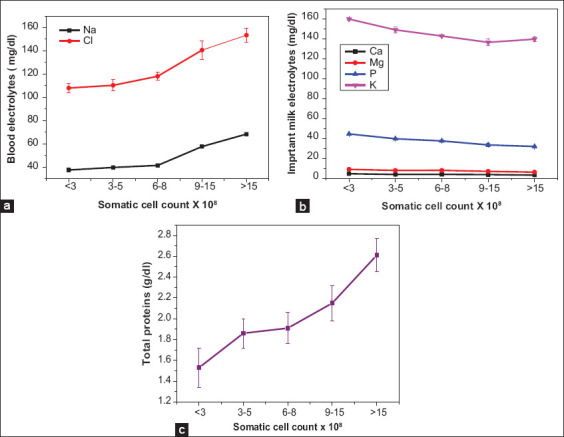
Correlation between somatic cell count and (a) blood electrolytes, (b) important electrolytes and (c) total proteins.

The average concentration of important electrolytes, including Ca, P, Mg, and K was reduced significantly along with a significant increase of the blood-borne electrolytes (Na and Cl) in cow’s milk with mastitis compared to those without (Table-4). Extreme changes were observed in the case of *Mycoplasma* mastitis, which was associated with elevated SCC compared to the non-*Mycoplasma* mastitis group. This reflects the contribution of mastitis severity to the influx of blood electrolytes into milk [16]. The total protein level shows a significant increase in mastitis compared to the non-mastitis groups, with a pronounced high level in *Mycoplasma* mastitis groups. As indicated in Figure-2, modification of milk electrolytes in addition to increased total proteins was found to be significantly correlated with the increase of SCC in milk.

**Table-4:** Electrolytes and total proteins in milk the different mastitis groups linked to SCC.

Mastitis groups	Blood electrolytes		Important electrolytes				Total protein (g/dL)
	
	Na (mg/dL)	Cl (mg/dL)	Ca (mg/dL)	P (mg/dL)	Mg (mg/dL)	K (mg/dL)	
Negative mastitis	37.50±0.95^a^	108.00±3.78^a^	4.58±0.19^a^	44.50±0.19^a^	9.00±0.176^a^	159.63±1.335^a^	1.53±0.19^a^
Non-*Mycoplasma* mastitis	39.67±0.92^a^	110.33±4.801^b^	3.83±0.22^b^	39.67±0.84^b^	7.90±0.228^b^	148.83±3.049^b^	1.86±0.14^b^
*Mycoplasma* mastitis	41.44±1.36^b^	140.63±7.958^c^	3.98±0.11^b^	37.47±1.106^b^	7.94±0.139^b^	142.67±1.130^b^	1.91±0.15^b^
	57.67±1.48^c^	153.50±5.948^c^	3.73±0.14^b^	33.50±1.49^c^	6.95±0.109^c^	136.33±3.174^c^	2.15±0.17^c^
	68.38±1.46^d^	108.00±3.78^d^	3.25±0.12^c^	31.88±0.64^c^	6.15±0.160^d^	139.50±2.57^c^	2.61±0.16^d^
Sig. level	0.001^#^	0.001^#^	0.004	0.001^#^	0.001^#^	0.001^#^	0.001^#^

# significant using ANOVA test at p*<*0.001. Letters with different superscripts are significantly different. Na=Sodium, Cl=Chloride, Ca=Calcium, p*=*Phosphorus, Mg=Magnesium, K=Potassium

To determine the antioxidants properties of milk in the examined dairy herd, the antioxidants parameters, including total antioxidant activity, MDA, and enzymatic antioxidants (GSH, SOD and catalase) were examined in the different mastitis groups versus the negative mastitis group. Table-5 showed a significantly higher MDA level in the three groups of *Mycoplasma* mastitis with a mean maximum of 1.99±0.055, compared to non-*Mycoplasma* mastitis and negative mastitis. In contrast, the mean GSH level (nM/mL) was significantly decreased in the negative mastitis group compared to the non-*Mycoplasma* mastitis. The maximum reached 6.23±0.236 in the *Mycoplasma* mastitis group E. SOD average concentrations (m/mL) also showed a significant decrease with an average level of 2.03±0.021 in non-*Mycoplasma* mastitis compared to the negative mastitis group. A severe decrease was observed in the three *Mycoplasma* mastitis groups with the lowest average in group E. The detected average of catalase in milk from healthy cows was 5.10±0.15, with a lower average detected in cows with non-*Mycoplasma* mastitis, while the level of reduction reached to an extreme degree in *Mycoplasma* mastitis Group E. The total antioxidant activity also revealed a lower average in *Mycoplasma* mastitis compared to non-mycoplasma and negative mastitis group.

**Table-5:** Evaluation of antioxidants activity in milk from the different mastitis groups.

Mastitis groups	Antioxidants parameter				

	MDA (nm/mL)	GSH (nm/mL)	SOD (μ/mL)	Catalase (µ/mL)	Total antioxidant activity (μmoL/L)
Negative	0.47±0.013^a^	13.00±0.378^a^	2.75±0.094^a^	5.10±0.151^a^	716.50±2.457^a^
Non-*Mycoplasma* mastitis	0.97±0.042^b^	9.33±0.211^b^	2.03±0.021^b^	4.67±0.056^b^	580.67±13.901^b^
*Mycoplasma* mastitis	1.01±0.041^b^	8.83±0.307^b^	1.98±0.060^b^	4.17±0.053^c^	581.50±10.570^b^
	1.39±0.066^c^	7.32±0.359^c^	1.77±0.053^c^	3.05±0.164^d^	508.56±15.903^abc^
	1.99±0.055^d^	6.23±0.236^d^	1.53±0.052^d^	3.60±0.113^e^	458.13±14.177^d^
Sig. level	0.001^#^	0.001^#^	0.001^#^	0.001^#^	0.001^#^

# significant using ANOVA test at p*<*0.001. ^a,b,c^and ^d^letter with different superscripts while were significantly different insignificant difference were observed between similar litter at p*<*0.05 using Duncan Multiple Range Test for comparative of means. SOD=Superoxide dismutase, MDA=Malondialdehyde, GSH=Glutathione peroxidase

**Figure-3:**
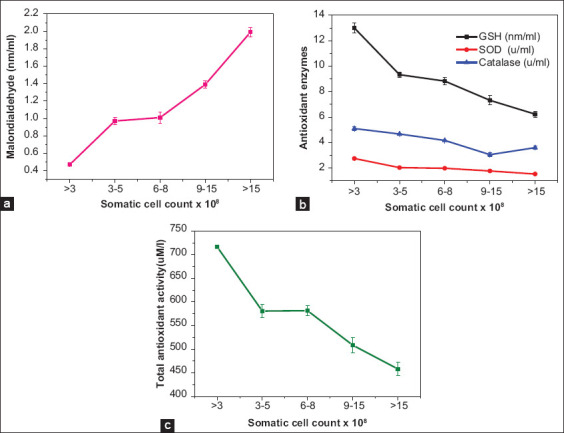
Clear correlation between the somatic cell count content in milk and (a) malondialdehyde concentration, (b) the antioxidant enzyme concentrations, (c) total antioxidant activity. Statistical significance was observed between tested samples (p=0.001).

Furthermore, the antioxidants parameters were plotted versus the SCC (Figure-3); this showed a positive correlation between MDA level and SCC (p=0.001), while the antioxidants enzymes and total antioxidants activity were correlated negatively with SCC (p=0.001).

### Detection of OCs pesticides residues in cow’s milk

Healthy cows (100) and mastitic cows (60) were screened for residues of 15 OCs compounds using GC, equipped with electron capture. Furthermore, the detection of OCs residues by GC analysis was confirmed in the positive milk samples by GC-mass spectrometry. The analysis confirmed that 45 (28.1%) out of 160 milk samples contained residues for at least one compound of OCs pesticides.

As shown in Table-6, the three analogs of dichlorodiphenyltrichloroethane (DDT) were detected in the analyzed milk samples; however, p, p´-dichlorodiphenyldichloroethane (p, p´ -DDD) was the most predominant OCs pesticides in mastitic milk (23.3%) in comparison to normal milk (12%). While p,p’-dichlorodiphenyldichloroethylene (p,p´ -DDE )was detected in 7% of mastitic cows and 3% of healthy cows. p,p´ -DDT was only found in one healthy cow at a range of 18 and one mastitic cow at a range of 21.0. The mastitic milk samples also showed residues for δ- and γ- isomers of hexachlorocyclohexane (HCH) with a detection rate of 6.7% and 0.6% and mean concentrations of 1.7±0.31 and 0.11, respectively. The α isomer of HCH along with methoxychlor and endosulfan were not detected in any of the examined milk samples. On the contrary, 20% of mastitic cows and 11% of healthy cows were found to pose hexachlorobenzene (HCB). Heptchlor epoxide residues were also determined in 15% of mastitic cows at a mean concentration of 11.2±0.8813, and 13% of healthy cows at a lower concentration.

**Table-6:** OCs pesticide residues (μg/L) determined in milk samples collected from a single herd of 160 dairy cows.

Pesticide name	Non-mastitic milk samples (n=100)			Mastitic milk samples (n=60)		
	
	No.	%	Mean±SE	No.	%	Mean±SE
HCB	11	11	11.5±0.41	12	20*	9.8±0.34
a-HCH	0	-	ND	0	-	ND
d-HCH	0	-	ND	4	6.7	1.7±0.31
g-HCH	0	-	ND	1	1.7	0.11
Aldrin	5	5	8.4±0.31	3	5	5.2±0.42
Endrin	4	4	9.1±0.54	6	10	8.1±0.46
Dieldrin	0	-	ND	2	3.4	2.0
Heptachlor	2	2	5.1	1	1.7	7.8
Hept. epoxide	13	13	9.5±0.83	9	15	11.2±0.88
c-Chlordane	2	2	6.3	3	5	7.2±0.93
Endosulfan	0	-	ND	0	-	ND
p, p' -DDE	3	3	2.2±0.18	4	7	2.3±0.28
p, p' -DDD	12	12	1.9±0.38	14	23.33	4.1±0.33
p, p' -DDT	1	1	18.0	1	1.7	21.0
Methoxychlor	0	0	ND	0	0	ND
Total OCP	19	19	26.7±19.53	26	43.3	38.31±24.62

OCs=Organochlorine, SE=Standard Error, OCP=Organocholerine pesticides, DDE=dichlorodiphenyldichloroethylene, DDD=Dichlorodiphenyldichloroethane, DDT=Dichlorodiphenyltrichloroethane, HCB=Hexachlorobenzene, HCH=Hexachlorocyclohexane

In addition, mastitic milk was found to be positive for aldrin (5%), endrin (10%) c-chlordane (5%), dieldrin (3.4%), and heptachlor (1.7%). The average detected concentration for each compound was as follows (mg/L): aldrin (5.2±0.42), endrin (8.1±0.46), dieldrin (2.0), C-chlordane (11.2±0.88), and heptachlor (7.2±0.93). Similarly, 5% of normal milk was found positive for aldrin, but a smaller percentage was positive for endrin. However, the analyzed normal milk samples were negative for dieldrin and only 2% contained residues for c-chlordane and heptachlor. The average concentrations detected in normal milk, as indicated in Table-4, appeared closely similar to those in mastitic milk. In total, 45 (28.5%) dairy cows in a single herd were detected posing OCs residues, with a higher incidence in mastitic cows (43.3%) than in healthy cows (19%).

## Discussion

Udder inflammatory diseases impact animal welfare and productivity, leading to severe economic losses worldwide. Various bacterial pathogens are known to have the ability to trigger udder inflammation, one of these, *Mycoplasma* mastitis has been reported to be widespread in dairies worldwide. *Mycoplasma* species, particularly *M. bovis* were the most transmissible pathogen in a dairy farm, causing a contagious form of mastitis [6]. Here, the overall incidence of mastitis recorded 37.5% and *Mycoplasma* mastitis accounts for 68.3% of the herd infection. The data identified *M. bovis* as the main responsible pathogen for mastitis infection in the single examined herd with 25.6% prevalence. The overall prevalence of *Mycoplasma* mastitis was 32.5% of the lactating cows, while only 5% of the dairy cows were diagnosed with non-*Mycoplasma* mastitis. This finding indicated an increased trend than previous estimates (8.96%) in Egypt during 2016 and 2019 [36]. In accordance with what recently established that *Mycoplasma* mastitis is increasing in prevalence worldwide, for example, a study in Israel reported annual growth in several positive herds for *M. bovis* mastitis during 2008 and 2014 [37]. Radaelli *et al*. [38] also demonstrated an outbreak of *Mycoplasma* mastitis affecting 55 cows in a dairy herd of 122 cows in Northern Italy. Dairies of South Australia were also reported a high prevalence of *Mycoplasma* mastitis (76.7%) resulted from coinfection with two or more *Mycoplasma* and *Acholeplasma* species [39].

Udder inflammatory diseases are associated with changes in biochemical constituents of milk as consequences of disturbance in the udder’s normal function by pathogens. The present work reported significant changes in the biochemical constituents of mastitis positive milk compared to negative, in agreement with other publications [39-43]. In line with the previous research, we observed marked alteration in milk electrolytes, including a significant rise of blood electrolytes (Na and Cl) and reduction of important electrolytes (Ca, P, Mg, and K). In these studies, changes were attributed to the reduced function of milk secretory cells and increased epithelium permeability, leading to an influx of blood electrolytes into milk [43,44]. Conversely, K is highly abundant in milk, but it leaches out of milk during udder inflammation. Total protein was also found to be elevated, which is in consistent with Petrovski [16] and Gráff and Miko [45], who linked this to leakage of blood proteins such as immunoglobulins and serum into milk as a response to immunological reactions during mastitis.

Measurement of somatic cell content in milk has frequently been used as a primary indicator of mastitis occurrence in dairies. In this work, SCC above 300K was considered an indication for mastitis, in agreement with Cobirka *et al*. [46]. It has been established in many peer-reviewed publications that elevated SCC is associated with marked changes in milk composition [47,48]. We also found that elevated SCC was consistently associated with *Mycoplasma* positivity and in turn, it appeared correlated with the elevated biochemical changes in milk. SCC has shown a positive correlation with blood electrolytes and a negative correlation with important milk electrolytes. This reflects the contribution of mastitis severity to influx of blood-borne- electrolytes into milk and leakage of important electrolytes from milk [49]. The total protein was also found to be positively correlated with the increase of milk SCC. Similar to our findings, a Turkish study on Holstein-Friesian cows demonstrated that level of protein and fat positively increased in milk with high SCC [50]. The positive correlation between the level of protein and SCC in milk has also been detected by Fernandes *et al*. [51].

According to the previous observations, milk with high somatic cell contents is highly infiltrated with polymorphonuclear (PMN) cells [52]. The fight of PMN against pathogenic organisms causing mastitis generates reactive oxygen species, and nitric oxide derivatives. Accumulation of these metabolites was shown to be associated with poor antioxidant activity, leading to oxidative stress. Oxidative stress is usually accompanied by altered enzymatic and non-enzymatic antioxidants and increased concentration of lipid peroxidation products (MDA). Increased SCC detected in this study was found to be associated with an increase of MDA concentration and depletion of antioxidant enzymes represented by GSH, SOD, and catalase. A study performed by Atakisi *et al*. [53] and Silanikove *et al*. [54] in cows and goats, respectively, observed depression of antioxidant activity. The two studies noted that SCC reflects severity of infection and directly correlated with low antioxidants activity. The determined relationship between high MDA concentration with an increase of SCC is similar to data presented by Suriyasathaporn *et al*. and Zigo *et al*. [55,56].

The changes in biochemical parameters in cow’s milk with clinical and subclinical mastitis were found to be associated with increased SCC, which is a successful indicator used in the diagnosis of subclinical mastitis in cows with no obvious signs of mammary gland inflammation. This suggests that biochemical parameters can serve as potential biomarkers for the early detection of mastitis. Various other studies have also reported biochemical changes associated with subclinical mastitis, for example, Qayyum *et al*. [57] observed changes in biochemical parameters in milk and blood from subclinical infected cattle and Andrei *et al*. [52] detected an alteration of antioxidant parameters in cows with subclinical mastitis. Furthermore, early detection of mastitis has been previously reported by tracking changes in systemic biochemical parameters of apparently healthy cows [56]. The degree of biochemical changes in milk depends on the severity and extent of udder infections. The impact of udder infections caused by bacterial pathogens other than *Mycoplasma* on milk composition has been well investigated; however, the impact of *Mycoplasma* mastitis on milk composition in comparison to non-*Mycoplasma* mastitis has not been completely clarified. We showed here that milk from udders with *Mycoplasma* mastitis tended to have greater SCC when compared to udders with non-*Mycoplasma* mastitis or a healthy udder. This is in consistent with previous studies that have linked the high somatic cell content to the prevalence of *Mycoplasma* mastitis [38,39,58]. We also showed that both *Mycoplasma* mastitis and non-*Mycoplasma* mastitis induced significant changes in the milk components. However, the leak of blood-borne electrolytes into milk along with depression of the important electrolytes, disturbance of antioxidant enzyme and total proteins was significantly greater in *Mycoplasma* mastitis. The biochemical changes observed in milk in the case of *Mycoplasma* mastitis in comparison to non-*Mycoplasma* mastitis highlights the serious impact of *Mycoplasma* infection on the physiology and function of udder. Our research has shown that modification of the biochemical composition of milk based on the types of mastitis was consistent with data in the literature [15].

A number of authors have studied the bacterial pathogens causing mastitis and discussed the factors that determine the susceptibility of cattle to udder infections [59-61]. The inherent genetic features and udder structure that govern the animal’s sensitivity to mastitis, hygiene measures related to milking and animal care have also been well investigated and identified [62-64]. Despite the advances and technologies applied in dairy industry to control the development of mastitis, the incidence of mastitis is still increasing. We argue in the present work that the continued growth of mastitis incidence in dairies might be related to dampening the herd immunity, as consequence of exposure of dairy cattle to environmental contaminants such as organic pesticides. Our data demonstrated an increased prevalence of clinical and subclinical mastitis (37.5%) in a single dairy herd, with 28.3% of lactating cows possessing OCs pesticides residues in their milk, which may be attributed to accidental exposure of dairy cows to contaminated feed.

The herd examined was grazing outdoors and was also allocated a daily meal of grains. Organic compounds with poor degradability and bio-accumulative properties have been extensively used in agriculture as herbicides and insecticides, resulting in their persistence in soil and water. OCs residues have recently been detected in surface and irrigation water in Egypt [12]. Furthermore, an increasing number of studies have reported OC residues in grains, used in animal feed. This suggests that the dairy herd may accumulate persistent organic compounds from contaminated feed, including grain, grass, and water [65,66]. OC pesticides are highly persistent lipophilic compounds, prone to bio-concentration in fat-rich tissues as the udder, with subsequent impact on milk purity [67,68]. Whilst their elimination is very slow through urine or stool, lactation is a major vehicle for excretion, while the recorded mean levels of OCs residues in milk were below the maximum residue limit (MRL) established by legislation [69]. The existence of OCs in the milk of dairy cows is an indicator of exposure to environmental OCs.

The total fraction of OCs in milk samples was significantly higher in cows with mastitis (43.3%) compared to those without (19% using Fischer Exact Probability Test). This suggests a potential correlation between susceptibility of dairy cattle to mastitis and exposure to OCs pesticides. Along with this, Sotnichenko *et a*l. [70] noted that spread of chemical impurities such as lipophilic pesticides in the environment has a high potential to contaminate the cattle feed and this might predispose dairy cattle to udder inflammatory diseases. Exposure of dairy cows to these toxic compounds is accompanied by a potentially damaging effect through dampening the innate immune response. This is reflected in an increasing amount of the data in the literature showing a correlation between pesticides exposure and adverse effect on the immune system [71,72].

## Conclusion

The present work highlights the serious impact of *Mycoplasma* mastitis on the dairy industry, where severity is reflected by elevated SCC associated with extensive biochemical changes in milk. This study also considered a potential association between exposure dairy cows to OCs pesticides and a higher incidence of herd mastitis. OCs exposure might increase the vulnerability of dairy cows to mastitis by decreasing the capability of their immune system to lunch an efficient immune defense against mastitis-causing pathogenic bacteria. This observation may lead to increased awareness of the negative indirect consequences on livestock productivity and public health that may result from the use of these hazardous compounds. However, further study is required to check the immune status of the animal regarding the level of pesticides in the animal’s blood or food, water, and soil.

## Authors’ Contributions

HRA, SFA, EK, RHA, SFA, and MA: Analysis and interpretation of data. HRA and EK: Drafted the manuscript. HRA, RHA, FAS, and MAS: Conception and design of the study. HRA and AK: Revised the manuscript critically. HRA, RHA, FAS, SFA, and MAS: Data collection and analysis. All authors read and approved the final manuscript.
